# Breakdown of a Nonlinear Stochastic Nipah Virus Epidemic Models through Efficient Numerical Methods

**DOI:** 10.3390/e23121588

**Published:** 2021-11-27

**Authors:** Ali Raza, Jan Awrejcewicz, Muhammad Rafiq, Muhammad Mohsin

**Affiliations:** 1 Department of Mathematics, Govt. Maulana Zafar Ali Khan Graduate College Wazirabad, Punjab Higher Education Department (PHED), Lahore 54000, Pakistan; alimustasamcheema@gmail.com; 2 Department of Automation, Biomechanics and Mechatronics, Lodz University of Technology, 1/15 Stefanowskiego St., 90-924 Lodz, Poland; jan.awrejcewicz@p.lodz.pl; 3 Department of Mathematics, Faculty of Sciences, University of Central Punjab, Lahore 54600, Pakistan; m.rafiq@ucp.edu.pk; 4 Department of Mathematics, Technische Universitat Chemnitz, 62, 09111 Chemnitz, Germany

**Keywords:** Nipah virus, stochastic model, numerical methods, stability analysis

## Abstract

**Background:** Nipah virus (NiV) is a zoonotic virus (transmitted from animals to humans), which can also be transmitted through contaminated food or directly between people. According to a World Health Organization (WHO) report, the transmission of Nipah virus infection varies from animals to humans or humans to humans. The case fatality rate is estimated at 40% to 75%. The most infected regions include Cambodia, Ghana, Indonesia, Madagascar, the Philippines, and Thailand. The Nipah virus model is categorized into four parts: susceptible (S), exposed (E), infected (I), and recovered (R). **Methods:** The structural properties such as dynamical consistency, positivity, and boundedness are the considerable requirements of models in these fields. However, existing numerical methods like Euler–Maruyama and Stochastic Runge–Kutta fail to explain the main features of the biological problems. **Results:** The proposed stochastic non-standard finite difference (NSFD) employs standard and non-standard approaches in the numerical solution of the model, with positivity and boundedness as the characteristic determinants for efficiency and low-cost approximations. While the results from the existing standard stochastic methods converge conditionally or diverge in the long run, the solution by the stochastic NSFD method is stable and convergent over all time steps. **Conclusions:** The stochastic NSFD is an efficient, cost-effective method that accommodates all the desired feasible properties.

## 1. Introduction

In September 1998, in a village near Ipoh City, Perak State, West Malaysia, a case was reported as having similar symptoms to Japanese B encephalitis (JE) virus; it was dealt with as a standard routine case. Still, cases continued to occur in the region until February 1999. They were ascribed to the JE virus, which had previously caused porcine-associated outbreaks in Malaysia and had been treated accordingly. It was later discovered in March 1999 by virologists from the University of Malaya that the cases were caused by a new virus that belongs to the family Paramyxoviridae, which does not include the JE virus. The virus was named the “Nipah Virus (NiV)”, after Kampung Sungai Nipah (Nipah river village), whose patient’s specimens yielded the first viral isolates. Nipah virus is a zoonotic virus, which means it spreads between animals and people. It belongs to the Paramyxoviridae family and, genetically, it is related to the Hendra Virus.

Flying foxes such as fruit bats are considered animal reservoirs for NiV. The outbreak of NiV infection started in September 1998 in a village near Ipoh City of Perak State, West Malaysia, which affected several areas just outside the town. In this epidemic, 27 patients were reported, which caused 15 deaths. Until March 1999, published sources quoted a prevalence of 265 cases of acute NiV encephalitis with 105 fatalities in Malaysia, giving a mortality of nearly 40%. In response to NiV, more than 1 million pigs were culled as it was discovered that sick pigs were significant carriers of the virus and further could transmit it to humans.

Furthermore, it was discovered that some of the pigs might have consumed partially eaten fruit by fruit bats that were affected by the virus. The virus also hit Singapore, where sick animals were imported from Malaysia during the NiV outbreak. The total number of cases reported in Singapore was 11, out of which one death was documented. These two countries have had no issue reported since 1999, but outbreaks continue to occur in Bangladesh and India. The same kind of virus emerged in India and its neighboring country Bangladesh in 2001 but it was not investigated until March 2003. As Bangladesh is a Muslim country where the pig industry is not present, the virus was able to spread through raw date palm sap. Later, it was observed that affected bats’ saliva, urine, and excreta might have mixed in the fluid, which resulted in the spread of NiV. In 2014, the virus emerged in the Philippines, causing human deaths and sudden deaths in several horses. It was thought that the virus was transmitted to humans by way of direct exposure to infected horses.

Tan et al. studied the incidence, distribution, and control of the Nipah virus [1]. Chua presented the outbreak of the Nipah virus in its origin country, Malaysia [2]. Chua et al. studied the viral infection in the first three infected people who were pig farmers [3]. Looi et al. investigated the need to understand the dynamics of the Nipah virus [4]. Sherrini et al. presented updates on the Nipah virus [5]. Lam et al. studied the spread of encephalitis (brain swelling disease) due to the Nipah virus [6]. Nicholas et al. presented the spread of the Nipah virus in Singapore among abattoir workers [7]. Chew et al. investigated the elements causing the spread of the Nipah virus among abattoir workers [8]. Yob et al. studied flying foxes such as bats as carriers of the Nipah virus in Peninsular, Malaysia [9]. Hsu et al. studied the re-emergence of the Nipah virus in Bangladesh [10]. Chadha et al. presented the spread of the Nipah virus in Siliguri, India [11]. Hughes et al. studied the transmission of Nipah virus infection in humans [12]. Chong et al. studied the Nipah virus and differences in the outbreaks of Malaysia and Bangladesh [13]. Clayton et al. predicted how the virus can transmit between humans and animals in Malaysia and Bangladesh [14]. Chua et al. studied the events that caused the emergence of the Nipah virus in Malaysia [15]. Sendow et al. analyzed in Sumatera, Indonesia, how the fruit bat was also the primary host of Nipah virus [16]. Mood et al. studied the viruses as biological warfare agents (BWA) and whether they can be created to demolish an area of choice [17]. Lam proved that the Nipah virus is not just a virus but a bioterrorist agent [18]. Satterfield et al. agreed that no licensed treatment is available up to date, but that vaccine research and development are still being carried out [19]. Sharma et al., in 2018, provided a review on the emerging and re-emerging of the Nipah virus [20]. Some notable models related to cervical cancer and many more diseases are presented in [21,22,23,24,25,26]. The well-known mathematical models in the sense of stochastic technique are presented in [27,28,29,30]. A lot of mathematical models have been studied with the help of different strategies as presented in [31,32]. It is that kind of model that exhibits a situation where randomness exists. In other words, a model for a process that possesses some uncertainty is a stochastic model. There are four significant stochastic models: parametric and non-parametric ways of modeling, modeling based on stochastic differential equations, modeling based on continuous time Markov chains, and modeling based on discrete-time Markov chains. The idea of stochastic differential equations was presented in 1942. Stochastic differential equations contribute an essential part to the composition of stochastic phenomena into the models. Due to the concept of SDEs, there has been a lot of development in different fields, including mechanics, biology, mathematics, chemistry, medicine, finance, physics, etc. The solution of SDEs is nowhere. The nonexistence of this solution is because of non-differential aspects of the Brownian motion. Thus, to study such kinds of differential equations, numerical approximations are applied. In addition, the stochastic representation of physical problems is close to the natural phenomena. The remaining types of studies for physical issues are not very close to nature. That is why we consider stochastic differential equations or stochastic models for study purposes. The rest of the paper is organized based on the following sections: In Section 2, the deterministic Nipah model’s formulation has fundamental properties. Section 3 deals with the stochastic model’s transition probabilities, positivity, boundedness and implementation methods, convergence, and comparative analysis. Finally, the conclusion is presented in Section 4.

## 2. Model Formulation

**Figure 1:**
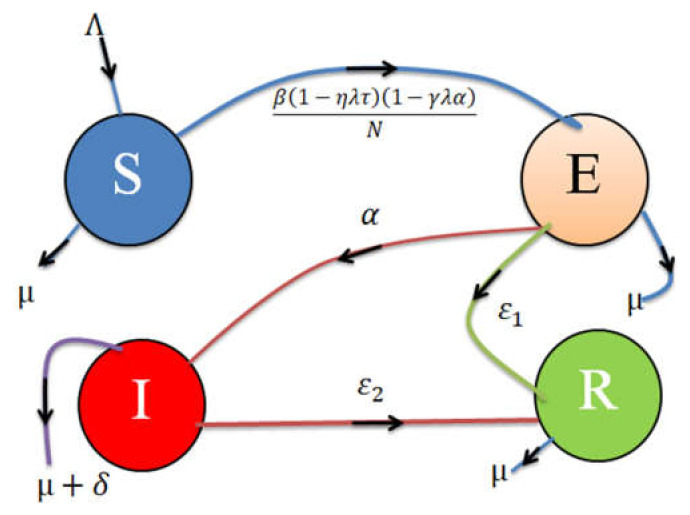
Flow map for the dynamics of the Nipah virus epidemic model.

At any time, the states of the model are described as follows: S(t) represents people who are susceptible to the Nipah virus; E(t) means people exposed to the Nipah virus but not infected; I(t) means people who are infected with the Nipah virus and can transmit the virus; R(t) means people recovered from the Nipah virus; Λ represents the number of people susceptible based on the birth rate; β represents the recruitment rate; α represents progression rate of infected people; δ represents the death rate because of disease; µ represents a natural rate of death; ε_1_ represents the recovery rate of exposed individuals due to awareness; ε_2_ represents the recovery rate of infected individuals due to treatment; η represents the number of people quarantined; τ represents the number of isolation centers available; γ represents increased personal hygiene due to public awareness; σ represents a rate of public awareness; λ means surveillance coverage. The systematic flow of Nipah virus disease is presented in Figure 1.

The nonlinear ordinary differential equations by using the law of mass action are as follows:

(1) $$S^{\prime }\left(t\right)=A-\frac{\beta \left(1-n\lambda \gamma \right)\left(1-\gamma \lambda \alpha \right)}{N}I\left(t\right)S\left(t\right)-\mu S\left(t\right),\;t\geq 0$$

(2) $$E^{\prime }\left(t\right)=\frac{\beta \left(1-n\lambda \gamma \right)\left(1-\gamma \lambda \alpha \right)}{N}I\left(t\right)S\left(t\right)-\alpha E\left(t\right)-\varepsilon_{1}E\left(t\right)-\mu E\left(t\right),\;t\geq 0$$

(3) $$I^{\prime }\left(t\right)=\alpha E\left(t\right)-\varepsilon_{2}I\left(t\right)-\delta I\left(t\right)-\mu I\left(t\right),\;t\geq 0$$

(4) $$R^{\prime }\left(t\right)=\varepsilon_{1}E\left(t\right)+\varepsilon_{2}I\left(t\right)-\mu R\left(t\right),\;t\geq 0$$

with nonnegative (initial) conditions *S* (0) ≥ 0, *E* (0) ≥ 0, *I* (0) ≥ 0, *R* (0) ≥ 0, and $S\left(t\right)+E\left(t\right)+I\left(t\right)+R\left(t\right)=N.$

### 2.1. Model Analysis

In this section, we will discuss the positivity and boundedness of solutions of the system (1)–(4) with initial conditions.

$$\chi =\left\{\left(S,\;E,\;I,\;R\right)\in R_{+}^{4}:N\left(t\right)\leq \frac{\Lambda }{\mu }\;,S\geq 0,E\geq 0,I\geq 0,\;R\geq 0\;\right\}$$

**Theorem** **1.** *The results of the system (1)–(4) with given initial conditions are positive for all*t≥0.

**Proof.**  By considering Equation (1),

$$\frac{\mathrm{dS}}{\mathrm{dt}}=\Lambda -\frac{\beta \left(1-n\lambda \gamma \right)\left(1-\gamma \lambda \alpha \right)}{N}IS-\mu S$$

$$\frac{\mathrm{dS}}{\mathrm{dt}}\geq -\frac{\beta \left(1-n\lambda \gamma \right)\left(1-\gamma \lambda \alpha \right)}{N}IS-\mu S$$

$$\frac{ds}{dt}\geq -\left(\frac{\beta \left(1-n\lambda \gamma \right)\left(1-\gamma \lambda \alpha \right)}{N}I+\mu \right)S$$

$$\int \frac{ds}{s}\geq \int -\left(\frac{\beta \left(1-n\lambda \gamma \right)\left(1-\gamma \lambda \alpha \right)}{N}I+\mu \right)dt$$

$$lns\geq \int -\left(\frac{\beta \left(1-n\lambda \gamma \right)\left(1-\gamma \lambda \alpha \right)}{N}I+\mu \right)dt$$

$$s\left(t\right)\geq s\left(0\right)e^{\int -\left(\frac{\beta \left(1-n\lambda \gamma \right)\left(1-\gamma \lambda \alpha \right)}{N}I+\mu \right)dt}\geq 0$$

Similarly, for Equations (2)–(4), we have:

$$E\left(t\right)\geq E\left(0\right)e^{\int -\left(\alpha +\varepsilon_{1}+\mu \right)dt}\geq 0$$

$$I\left(t\right)\geq I\left(0\right)e^{\int -\left(\varepsilon_{2}+\delta +\mu \right)dt}\geq 0$$

$R\left(t\right)\geq R\left(0\right)e^{\int -\mu dt}\geq 0$ as desired. □

**Theorem** **2.** *The solutions*$\left(S,E,I,R\right)\epsilon R_{+}^{4}$*of the system (1)–(4) are bounded at any time and*$\underset{it\to \infty }{lim}Sup\;N\left(t\right)\leq \frac{\Lambda }{\mu }$.

**Proof.**  By considering the population function as follows:

$$N=S\left(t\right)+E\left(t\right)+I\left(t\right)+R\left(t\right)$$

$$\frac{dN}{dt}=\frac{dS}{dt}+\frac{dE}{dt}+\frac{dI}{dt}+\frac{dR}{dt},\;t\geq 0$$

$$\frac{dN}{dt}\leq \Lambda -\mu \left[S\left(t\right)+E\left(t\right)+I\left(t\right)+R\left(t\right)\right]$$

$$\frac{dN}{dt}\leq \Lambda -\mu N$$

$$\frac{dN}{dt}+\mu N\leq \Lambda$$

$$N\left(t\right)\leq \frac{\Lambda }{\mu }+e^{-\mu t}\;N\left(0\right)$$

$$N\left(t\right)\leq \frac{\Lambda }{\mu }+N\left(0\right)e^{-\mu t}$$

For large t*→∞*

$\underset{t\to \infty }{lim}Sup\;N\left(t\right)\leq \frac{\Lambda }{\mu }$, as desired. □

### 2.2. Equilibria

The system (1)–(4) admits two types of equilibria as follows: disease-free equilibrium = $\left(\frac{\Lambda }{\mu },0,0,0\right)$ and endemic equilibrium = ($S^{*}$,$\;E^{*}$,$\;I^{*}$,$\;R^{*}$)

$$S^{*}=\frac{N(\varepsilon_{2}+\delta +\mu )\left(\alpha +\varepsilon_{1}+\mu \right)}{\alpha \beta \left(1-\eta \lambda \tau \right)\left(1-\gamma \lambda \sigma \right)},\;E^{*}=\frac{\Lambda \alpha \beta \left(1-\eta \lambda \tau \right)\left(1-\gamma \lambda \sigma \right)(\varepsilon_{2}+\delta +\mu )-\mu N\left(\alpha +\varepsilon_{1}+\mu \right){\left(\varepsilon_{2}+\delta +\mu \right)}^{2}}{\alpha \beta \left(1-\eta \lambda \tau \right)\left(1-\gamma \lambda \sigma \right)(\varepsilon_{2}+\delta +\mu )\left(\alpha +\varepsilon_{1}+\mu \right)},$$

$$I^{*}=\frac{\Lambda \alpha \beta \left(1-\eta \lambda \tau \right)\left(1-\gamma \lambda \sigma \right)-\mu N(\varepsilon_{2}+\delta +\mu )\left(\alpha +\varepsilon_{1}+\mu \right)}{\beta \left(1-\eta \lambda \tau \right)\left(1-\gamma \lambda \sigma \right)(\varepsilon_{2}+\delta +\mu )\left(\alpha +\varepsilon_{1}+\mu \right)}$$

$$R^{*}=\frac{\Lambda \alpha \beta \varepsilon_{1}\left(1-\eta \lambda \tau \right)\left(1-\gamma \lambda \sigma \right)(\varepsilon_{2}+\delta +\mu )-\mu N\varepsilon_{1}\left(\alpha +\varepsilon_{1}+\mu \right){\left(\varepsilon_{2}+\delta +\mu \right)}^{2}+\Lambda \alpha \alpha \beta \varepsilon_{2}\left(1-\eta \lambda \tau \right)\left(1-\gamma \lambda \sigma \right)-\varepsilon_{2}\mu N\alpha (\varepsilon_{2}+\delta +\mu )\left(\alpha +\varepsilon_{1}+\mu \right)}{\alpha \beta \mu \left(1-\eta \lambda \tau \right)\left(1-\gamma \lambda \sigma \right)(\varepsilon_{2}+\delta +\mu )\left(\alpha +\varepsilon_{1}+\mu \right)}.$$

### 2.3. Reproduction Number

The next-generation matrix method is presented for the system (1)–(4). We calculate two types of matrices. One is a transition matrix, and the second is a transmission matrix, as follows:

$$\left[\begin{array}{c} E^{\prime }\\ I^{\prime }\\ R^{\prime } \end{array}\right]=\left[\begin{array}{ccc} 0 & \frac{\beta \left(1-\eta \lambda \tau \right)\left(1-\gamma \lambda \sigma \right)S}{N} & 0\\ 0 & 0 & 0\\ 0 & 0 & 0 \end{array}\right]\left[\begin{array}{c} E\\ I\\ R \end{array}\right]-\left[\begin{array}{ccc} \left(\alpha +\varepsilon_{1}+\mu \right) & 0 & 0\\ -\alpha & \left(\varepsilon_{2}+\delta +\mu \right) & 0\\ -\varepsilon_{1} & -\varepsilon_{2} & \mu \end{array}\right]\left[\begin{array}{c} E\\ I\\ R \end{array}\right]$$

where $F=\left[\begin{array}{ccc} 0 & \frac{\beta \left(1-\eta \lambda \tau \right)\left(1-\gamma \lambda \sigma \right)S}{N} & 0\\ 0 & 0 & 0\\ 0 & 0 & 0 \end{array}\right]$, $G=\left[\begin{array}{ccc} \left(\alpha +\varepsilon_{1}+\mu \right) & 0 & 0\\ -\alpha & \left(\varepsilon_{2}+\delta +\mu \right) & 0\\ -\varepsilon_{1} & -\varepsilon_{2} & \mu \end{array}\right]$ are the transition and transmission matrices, respectively.

$$FG^{-1}=\left[\begin{array}{ccc} \frac{\alpha \beta \mu \left(1-\eta \lambda \tau \right)\left(1-\gamma \lambda \sigma \right)S}{N\mu \left(\alpha +\varepsilon_{1}+\mu \right)\left(\varepsilon_{2}+\delta +\mu \right)} & \frac{\mu \beta \left(\alpha +\varepsilon_{1}+\mu \right)\left(1-\eta \lambda \tau \right)\left(1-\gamma \lambda \sigma \right)S}{N\mu \left(\alpha +\varepsilon_{1}+\mu \right)\left(\varepsilon_{2}+\delta +\mu \right)} & 0\\ 0 & 0 & 0\\ 0 & 0 & 0 \end{array}\right]$$

$$\left|\begin{array}{c} FG^{-1}-\lambda \end{array}\right|=\left|\begin{array}{ccc} \frac{\alpha \beta \left(1-\eta \lambda \tau \right)\left(1-\gamma \lambda \sigma \right)S}{N\left(\alpha +\varepsilon_{1}+\mu \right)\left(\varepsilon_{2}+\delta +\mu \right)}-\lambda & \frac{\beta \left(1-\eta \lambda \tau \right)\left(1-\gamma \lambda \sigma \right)S}{N\left(\varepsilon_{2}+\delta +\mu \right)} & 0\\ 0 & 0-\lambda & 0\\ 0 & 0 & 0-\lambda \end{array}\right|=0$$

The spectral radius of the $FG^{-1}$, called the reproduction number, is as follows:

$$R_{0}=\frac{\alpha \beta \left(1-\eta \lambda \tau \right)\left(1-\gamma \lambda \sigma \right)\Lambda }{N\left(\alpha +\varepsilon_{1}+\mu \right)\left(\varepsilon_{2}+\delta +\mu \right)\mu }$$

### 2.4. Stability Results

**Theorem** **3.** *The disease-free equilibrium =*$\left(\frac{\Lambda }{\mu },0,0,0\right)$*is locally asymptotically stable (LAS) when*$R_{0}$*< 1*.

**Proof.**  The Jacobian matrix at the disease-free equilibrium is as follows:

$$\left|\begin{array}{c} J\left(\frac{\Lambda }{\mu },0,0,0\right)-\lambda I \end{array}\right|=\left|\begin{array}{cccc} -\mu -\lambda & 0 & -\frac{\beta \left(1-\eta \lambda \tau \right)\left(1-\gamma \lambda \sigma \right)\frac{\Lambda }{\mu }}{N} & 0\\ 0 & -\left(\alpha +\varepsilon_{1}+\mu \right)-\lambda & \frac{\beta \left(1-\eta \lambda \tau \right)\left(1-\gamma \lambda \sigma \right)\frac{\Lambda }{\mu }}{N} & 0\\ 0 & \alpha & -\left(\varepsilon_{2}+\delta +\mu \right)-\lambda & 0\\ 0 & \varepsilon_{1} & \varepsilon_{2} & -\mu -\lambda \end{array}\right|\;=0$$

$$\lambda_{1}=-\mu <0,\;\lambda_{2}=-\mu <0$$

$$\left[\left\{-\left(\alpha +\varepsilon_{1}+\mu \right)-\lambda \right\}\left\{-\left(\varepsilon_{2}+\delta +\mu \right)-\lambda \right\}-\alpha \left\{\frac{\beta \left(1-\eta \lambda \tau \right)\left(1-\gamma \lambda \sigma \right)\frac{\Lambda }{\mu }}{N}\right\}\right]=0$$

$$\left(A+\lambda \right)\left(B+\lambda \right)-C=0$$

$$AB+A\lambda +B\lambda +\lambda^{2}-C=0$$

$$\lambda^{2}+\left(A+B\right)\lambda +AB-C=0$$

where $A\;=\left(\alpha +\varepsilon_{1}+\mu \right)$, $B\;=\left(\varepsilon_{2}+\delta +\mu \right)$, $\;C\;=\alpha \left\{\frac{\beta \left(1-\eta \lambda \tau \right)\left(1-\gamma \lambda \sigma \right)\frac{\Lambda }{\mu }}{N}\right\}$.

By using the Routh–Hurwitz criteria of 2nd order $\left(A+B\right)>0$, AB−C>0, if:

$$R_{0}=\frac{\alpha \beta \left(1-\eta \lambda \tau \right)\left(1-\gamma \lambda \sigma \right)\Lambda }{N\left(\alpha +\varepsilon_{1}+\mu \right)\left(\varepsilon_{2}+\delta +\mu \right)\mu }<1$$

Hence, disease-free equilibrium is local asymptotically stable (LAS). □

**Theorem** **4.** *The endemic equilibrium = (*$S^{*}$, $E^{*}$, $I^{*}$, $R^{*}$) is locally asymptotically stable (LAS) when$R_{0}$*> 1*.

**Proof.**  The Jacobian matrix at the endemic equilibrium is as follows:

$$J=\left[\begin{array}{cccc} -\frac{\beta \left(1-\eta \lambda \tau \right)\left(1-\gamma \lambda \sigma \right)I^{*}}{N}-\mu & 0 & -\frac{\beta \left(1-\eta \lambda \tau \right)\left(1-\gamma \lambda \sigma \right)S^{*}}{N} & 0\\ \frac{\beta \left(1-\eta \lambda \tau \right)\left(1-\gamma \lambda \sigma \right)I^{*}}{N} & -\left(\alpha +\varepsilon_{1}+\mu \right) & \frac{\beta \left(1-\eta \lambda \tau \right)\left(1-\gamma \lambda \sigma \right)S^{*}}{N} & 0\\ 0 & \alpha & -\left(\varepsilon_{2}+\delta +\mu \right) & 0\\ 0 & \varepsilon_{1} & \varepsilon_{2} & -\mu \end{array}\right]$$

$$\left|\begin{array}{c} J\left(S^{*},\;E^{*},\;I^{*},\;R^{*}\right)-\lambda^{*}I \end{array}\right|=\left|\begin{array}{cccc} a_{1}-\lambda^{*} & 0 & a_{2} & 0\\ a_{3} & a_{4}-\lambda^{*} & a_{5} & 0\\ 0 & \alpha & a_{6}-\lambda^{*} & 0\\ 0 & \varepsilon_{1} & \varepsilon_{2} & -\mu -\lambda^{*} \end{array}\right|=0$$

$$\lambda^{*}{}_{1}=-\mu <0$$

$$\left|\begin{array}{c} J\left(S^{*},\;E^{*},\;I^{*},\;R^{*}\right)-\lambda^{*}I \end{array}\right|=\left|\begin{array}{ccc} a_{1}-\lambda^{*} & 0 & a_{2}\\ a_{3} & a_{4}-\lambda^{*} & a_{5}\\ 0 & \alpha & a_{6}-\lambda^{*} \end{array}\right|=0$$

$$(a_{1}-\lambda^{*})\left[\left(a_{4}-\lambda^{*}\right)\left(a_{6}-\lambda^{*}\right)-\alpha a_{5}]+a_{2}[\alpha a_{3}\right]=0$$

$$\lambda^{*}{}^{3}+(a_{4}+a_{6}-a_{1})\lambda^{*}{}^{2}-(a_{1}a_{4}-a_{1}a_{6}-a_{4}a_{6}-a_{5}\alpha )\lambda^{*}+a_{1}a_{4}a_{6}-a_{1}a_{5}\alpha +a_{2}a_{3}\alpha =0$$

where $a_{1}=\frac{-\beta \left(1-\eta \lambda \tau \right)\left(1-\gamma \lambda \sigma \right)I^{*}}{N}-\mu$, $a_{2}=\frac{-\beta \left(1-\eta \lambda \tau \right)\left(1-\gamma \lambda \sigma \right)S^{*}}{N}$, $a_{3}=\frac{\beta \left(1-\eta \lambda \tau \right)\left(1-\gamma \lambda \sigma \right)I^{*}}{N}$, $a_{4}=-\left(\alpha +\varepsilon_{1}+\mu \right)$, $a_{5}=\frac{\beta \left(1-\eta \lambda \tau \right)\left(1-\gamma \lambda \sigma \right)S^{*}}{N}$ and $a_{6}=-\left(\varepsilon_{2}+\delta +\mu \right)$.

By applying the Routh–Hurwitz Criterion for the 3rd order, $(a_{4}+a_{6}-a_{1})>0,\;(a_{1}a_{4}a_{6}-a_{1}a_{5}\alpha +a_{2}a_{3}\alpha )>0,\;$ and $\left(a_{4}+a_{6}-a_{1})(a_{1}a_{4}-a_{1}a_{6}-a_{4}a_{6}-a_{5}\alpha )>(a_{1}a_{4}a_{6}-a_{1}a_{5}\alpha +a_{2}a_{3}\alpha \right)$, if $R_{0}>1$. Hence, the given system is locally asymptotically stable. □

**Definition** **1.** *Probability Space [33]: A* probability space *is a three-tuple*, $\left(S,\;F,P\right)$*, in which the three components are: Sample space: A nonempty set* S *called the* sample space*, which represents all possible outcomes; Event space: A collection* F *of subsets of* S *called the* event space*. If* S *is discrete, then usually* F=pow(S). *If* S *is continuous, then* F *is usually a sigma-algebra on* S*, and Probability function: A function,* P:F→ℝ*, that assigns probabilities to the events in* F *. This will sometimes be referred to as a* probability distribution *over* S*. The probability function,* P *must satisfy several basic axioms:*

*(i)*  $P\left(E_{1}\right)\geq 0,\forall E_{1}\epsilon F.$
*(ii)*  $P\left(S\right)=1$.
*(iii)*  $P\left(E_{1}+E_{2}\right)=P\left(E_{1}\right)+P\left(E_{2}\right),$$E_{1}\cap E_{2}=\emptyset ,\;\forall \;E_{1},\;E_{2}\epsilon F.$

**Definition** **2.** *Brownian Motion: The Brownian motion process*$B_{t}$*is categorized by four facts* [32]:

*(i)*  $B_{0}=0$.
*(i)*  $B_{t}$*must be continuous, the event happens with probability one. The sample trajectories*$t\to B_{t}$*are continuous with probability one*.
*(iii)*  *For any finite sequence of times*$t_{1}<t_{2}<t_{3}\ldots <t_{n}$. *The following paths*$B_{t_{1}}-B_{t_{o}},B_{t_{2}}-B_{t_{1}},B_{t_{3}}-B_{t_{2}}\ldots ,B_{t_{n}}-B_{t_{n-1}}$*are independent*.
*(iv)*  *For any times*$0\leq s\leq t,\;B_{t}-B_{s}$*is normally distributed with mean zero and variance is*t−s. *In particular, we say that*$expectation\;\left[B_{t}-B_{s}\right]=0\;and\;variance\;\left[B_{t}-B_{s}\right]=t-s.\;$

## 3. Stochastic Model

We consider a vector $C\left(t\right)={\left[S\left(t\right),\;E\left(t\right),\;I\left(t\right),\;R\left(t\right)\right]}^{T}$ of stochastic differential equations (SDE’s) of the Nipah virus epidemic model. We want to calculate $E^{*}\left[\Delta C\left(t\right)\right]$ and variance ${\;E}^{*}\left[\Delta C\left(t\right)\;\Delta C{\left(t\right)}^{T}\right]$ expectations. To find the likely changes and their related transition probabilities (see Table 1) [34].

$$\mathrm{Expectation}=E^{*}\left[\Delta C\right]=\sum_{i=1}^{10}P_{i}{\left(\Delta C\right)}_{i}\;=P_{1}{\left(\Delta C\right)}_{1}+P_{2}{\left(\Delta C\right)}_{2}+P_{3}{\left(\Delta C\right)}_{3}+P_{4}{\left(\Delta C\right)}_{4}+P_{5}{\left(\Delta C\right)}_{5}+P_{6}{\left(\Delta C\right)}_{6}+P_{7}{\left(\Delta C\right)}_{7}+P_{8}{\left(\Delta C\right)}_{8}+P_{9}{\left(\Delta C\right)}_{9}+P_{10}{\left(\Delta C\right)}_{10}\;=\left[\begin{array}{c} P_{1}-P_{2}-P_{3}\\ P_{2}-P_{4}-P_{5}-P_{6}\\ P_{4}-P_{7}-P_{8}-P_{9}\\ P_{5}+P_{7}-P_{10} \end{array}\right]\;=\left[\begin{array}{c} \Lambda -\frac{\beta \left(1-\eta \lambda \tau \right)\left(1-\gamma \lambda \sigma \right)IS}{N}-\mu S\;\\ \frac{\beta \left(1-\eta \lambda \tau \right)\left(1-\gamma \lambda \sigma \right)IS}{N}-\alpha E-\varepsilon_{1}E-\mu E\\ \;\alpha E-\varepsilon_{2}I-\delta I-\mu I\\ \;\varepsilon_{1}E+\varepsilon_{2}I-\mu R \end{array}\right]\;\Delta t$$

$$\mathrm{Variance}=E^{*}\left[\Delta C\;\Delta C^{T}\right]=\sum_{I\;=1}^{10}P_{i}[{\left(\Delta C\right)}_{i}]{\left[{\left(\Delta C\right)}_{i}\right]}^{T}\;=P_{1}{\left(\Delta C\right)}_{1}{\left[{\left(\Delta C\right)}_{1}\right]}^{t}+P_{2}{\left(\Delta C\right)}_{2}{\left[{\left(\Delta C\right)}_{2}\right]}^{t}+P_{3}{\left(\Delta C\right)}_{3}{\left[{\left(\Delta C\right)}_{3}\right]}^{t}+\ldots +P_{10}{\left(\Delta C\right)}_{10}{\left[{\left(\Delta C\right)}_{10}\right]}^{t}\;=\left[\begin{array}{cccc} P_{1}+P_{2}+P_{3} & -P_{2} & 0 & 0\\ -P_{2} & P_{2}+P_{4}+P_{5}+P_{6} & -P_{4} & -P_{5}\\ 0 & -P_{4} & P_{4}+P_{7}+P_{8}+P_{9} & -P_{7}\\ 0 & -P_{5} & -P_{7} & P_{5}+P_{7}+P_{10} \end{array}\right]\Delta t\;=\left[\begin{array}{cccc} W_{11} & W_{12} & W_{13} & W_{14}\\ W_{21} & W_{22} & W_{23} & W_{24}\\ W_{31} & W_{32} & W_{33} & W_{34}\\ W_{41} & W_{42} & W_{43} & W_{44} \end{array}\right]\Delta t$$

where: 

$W_{11}=\;\Lambda +\frac{\beta \left(1-\eta \lambda \tau \right)\left(1-\gamma \lambda \sigma \right)IS}{N}+\mu S,{\;W}_{12}=-\frac{\beta \left(1-\eta \lambda \tau \right)\left(1-\gamma \lambda \sigma \right)IS}{N},{\;W}_{13}=0,W_{14}=0$,$W_{21}=-\frac{\beta \left(1-\eta \lambda \tau \right)\left(1-\gamma \lambda \sigma \right)IS}{N},{\;W}_{22}=\frac{\beta \left(1-\eta \lambda \tau \right)\left(1-\gamma \lambda \sigma \right)IS}{N}+\alpha E+\varepsilon_{1}E+\mu E,{\;W}_{23}=-\alpha E,W_{24}=-\varepsilon_{1}E\;$, $W_{31}=0,{\;W}_{32}=-\alpha E,{\;W}_{33}=\alpha E+\varepsilon_{2}I+\delta I+\mu I\;,W_{34}=-\varepsilon_{2}I\;,{\;W}_{41}=0\;,{\;W}_{42}=-\varepsilon_{1}E\;,{\;W}_{43}=-\varepsilon_{2}I\;,{\;W}_{44}=\varepsilon_{1}E+\varepsilon_{2}I+\mu R\;$

$$\mathrm{Drift}\;=\;G\left(C,t\right)=\frac{E^{*}\left[\Delta C\right]}{\Delta t}=\left[\begin{array}{c} \Lambda -\frac{\beta \left(1-\eta \lambda \tau \right)\left(1-\gamma \lambda \sigma \right)IS}{N}-\mu S\;\\ \frac{\beta \left(1-\eta \lambda \tau \right)\left(1-\gamma \lambda \sigma \right)IS}{N}-\alpha E-\varepsilon_{1}E-\mu E\\ \;\alpha E-\varepsilon_{2}I-\delta I-\mu I\\ \varepsilon_{1}E+\varepsilon_{2}I-\mu R \end{array}\right]\;\Delta t$$

$$\mathrm{Diffusion}\;=\;H\left(C,t\right)=\sqrt{\frac{E^{*}\left[\Delta C\;\Delta C^{T}\right]}{\Delta t}}=\sqrt{\;\left[\begin{array}{cccc} W_{11} & W_{12} & W_{13} & W_{14}\\ W_{21} & W_{22} & W_{23} & W_{24}\\ W_{31} & W_{32} & W_{33} & W_{34}\\ W_{41} & W_{42} & W_{43} & W_{44} \end{array}\right]}$$

$$\mathrm{Diffusion}=\;\sqrt{\left[\begin{array}{cccc} \;\Lambda +\frac{\beta \left(1-\eta \lambda \tau \right)\left(1-\gamma \lambda \sigma \right)IS}{N}+\mu S & -\frac{\beta \left(1-\eta \lambda \tau \right)\left(1-\gamma \lambda \sigma \right)IS}{N} & 0 & 0\\ -\frac{\beta \left(1-\eta \lambda \tau \right)\left(1-\gamma \lambda \sigma \right)IS}{N} & \frac{\beta \left(1-\eta \lambda \tau \right)\left(1-\gamma \lambda \sigma \right)IS}{N}+\alpha E+\varepsilon_{1}E+\mu E & -\alpha E & -\varepsilon_{1}E\\ 0 & -\alpha E & \;\alpha E+\varepsilon_{2}I+\delta I+\mu I & -\varepsilon_{2}I\\ 0 & -\varepsilon_{1}E\; & -\varepsilon_{2}I & \varepsilon_{1}E+\varepsilon_{2}I+\mu R \end{array}\right]}$$

The stochastic differential equations (SDEs) of pine wilt epidemic model (1)–(4) can be written as

(5) $$\mathrm{dC}\left(t\right)=\;G\left(C,t\right)\mathrm{dt}+H\left(C,t\right)dB\left(t\right)$$

or

(6) $$\begin{array}{l} d\left[\begin{array}{c} M\\ N\\ O\\ P \end{array}\right]\\ =\left[\begin{array}{c} \Lambda -\frac{\beta \left(1-\eta \lambda \tau \right)\left(1-\gamma \lambda \sigma \right)IS}{N}-\mu S\\ \frac{\beta \left(1-\eta \lambda \tau \right)\left(1-\gamma \lambda \sigma \right)IS}{N}-\alpha E-\varepsilon_{1}E-\mu E\\ \;\alpha E-\varepsilon_{2}I-\delta I-\mu I\\ \varepsilon_{1}E+\varepsilon_{2}I-\mu R \end{array}\right]\mathrm{dt}\\ +\sqrt{\left[\begin{array}{cccc} \Lambda +\frac{\beta \left(1-\eta \lambda \tau \right)\left(1-\gamma \lambda \sigma \right)IS}{N}+\mu S & -\frac{\beta \left(1-\eta \lambda \tau \right)\left(1-\gamma \lambda \sigma \right)IS}{N} & 0 & 0\\ -\frac{\beta \left(1-\eta \lambda \tau \right)\left(1-\gamma \lambda \sigma \right)IS}{N} & \frac{\beta \left(1-\eta \lambda \tau \right)\left(1-\gamma \lambda \sigma \right)IS}{N}+\alpha E+\varepsilon_{1}E+\mu E & -\alpha E & -\varepsilon_{1}E\\ 0 & -\alpha E & \alpha E+\varepsilon_{2}I+\delta I+\mu I & -\varepsilon_{2}I\\ 0 & -\varepsilon_{1}E & -\varepsilon_{2}I & \varepsilon_{1}E+\varepsilon_{2}I+\mu R \end{array}\right]}dB\left(t\right) \end{array}$$

with initial conditions $C\left(0\right)=C_{o}={\left[0.5,0.3,0.2,0.1\right]}^{T}\;,\;0\leq t\leq C\;\mathrm{and}\;B\left(t\right)$ is the Brownian motion.

### 3.1. Euler–Maruyama Method

**Figure 2:**
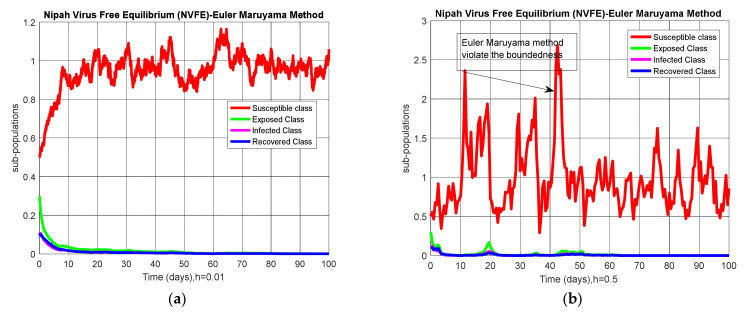
(**a**) Behavior of sub-populations at NVFE when h=0.01; (**b**) behavior of sub-populations at NVFE when h=0.5.

The Euler–Maruyama method is used to determine the numerical result of Equation (6) by using the parameters’ values given in Table 2 and Figure 2 to represent the data curation of the Nipah virus graphically.

The Euler–Maruyama method of stochastic differential Equation (6) is as follows:

$$C_{n+1}=C_{n}+f\left(C_{n},t\right)\Delta t+L\left(C_{n},t\right)dB\left(t\right)$$

(7) $$\begin{array}{l} \left[\begin{array}{c} M^{n+1}\\ N^{n+1}\\ O^{n+1}\\ P^{n+1}\\ Q^{n+1}\\ R^{n+1} \end{array}\right]\\ =\left[\begin{array}{c} M^{n}\\ N^{n}\\ O^{n}\\ P^{n}\\ Q^{n}\\ R^{n} \end{array}\right]+\left[\left[\begin{array}{c} \Lambda -\frac{\beta \left(1-\eta \lambda \tau \right)\left(1-\gamma \lambda \sigma \right)I^{n}S^{n}}{N}-\mu S^{n}\;\\ \frac{\beta \left(1-\eta \lambda \tau \right)\left(1-\gamma \lambda \sigma \right)I^{n}S^{n}}{N}-\alpha E^{n}-\varepsilon_{1}E^{n}-\mu E^{n}\\ \;\alpha E^{n}-\varepsilon_{2}I^{n}-\delta I^{n}-\mu I^{n}\\ \varepsilon_{1}E^{n}+\varepsilon_{2}I^{n}-\mu R^{n} \end{array}\right]\right]\Delta t\\ +\sqrt{\begin{array}{cccc} \Lambda +\frac{\beta \left(1-\eta \lambda \tau \right)\left(1-\gamma \lambda \sigma \right)I^{n}S^{n}}{N}+\mu S^{n} & -\frac{\beta \left(1-\eta \lambda \tau \right)\left(1-\gamma \lambda \sigma \right)I^{n}S^{n}}{N} & 0 & 0\\ -\frac{\beta \left(1-\eta \lambda \tau \right)\left(1-\gamma \lambda \sigma \right)I^{n}S^{n}}{N} & \frac{\beta \left(1-\eta \lambda \tau \right)\left(1-\gamma \lambda \sigma \right)I^{n}S^{n}}{N}+\alpha E^{n}+\varepsilon_{1}E^{n}+\mu E^{n} & -\alpha E^{n} & -\varepsilon_{1}E^{n}\\ 0 & -\alpha E^{n} & \alpha E^{n}+\varepsilon_{2}I^{n}+\delta I^{n}+\mu I^{n} & -\varepsilon_{2}I^{n}\\ 0 & -\varepsilon_{1}E^{n} & -\varepsilon_{2}I^{n} & \varepsilon_{1}E^{n}+\varepsilon_{2}I^{n}+\mu R^{n} \end{array}}\Delta B_{n} \end{array}$$

**Figure 3:**
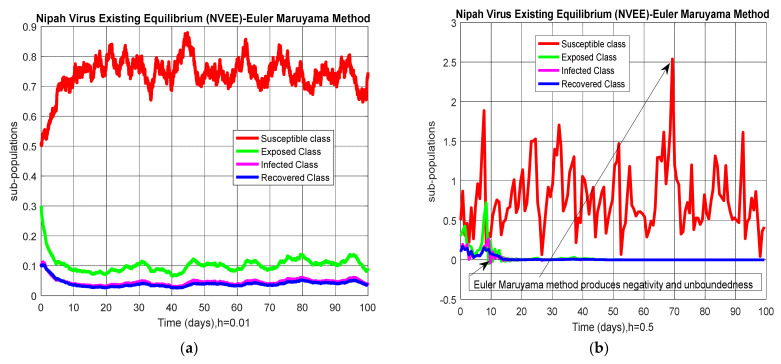
(**a**) Behavior of sub-populations at NVEE when h=0.01; (**b**) behavior of sub-populations at NVEE when h=0.5.

The graphical behavior of the Euler–Maruyama scheme for both equilibria is presented as shown in Figure 2 and Figure 3.

### 3.2. Non-Parametric Perturbation

In this section we introduce the non-parametric parameter into the system (1)–(4) as follows [35,36]:

(8) $$dS\left(t\right)=\left(A-\frac{\beta \left(1-n\lambda \gamma \right)\left(1-\gamma \lambda \alpha \right)}{N}I\left(t\right)S\left(t\right)-\mu S\left(t\right)\right)dt+\sigma_{1}S\left(t\right)dB\left(t\right)\;,\;t\geq 0$$

(9) $$dE\left(t\right)=\left(\frac{\beta \left(1-n\lambda \gamma \right)\left(1-\gamma \lambda \alpha \right)}{N}I\left(t\right)S\left(t\right)-\alpha E\left(t\right)-\varepsilon_{1}E\left(t\right)-\mu E\left(t\right)\right)dt+\sigma_{2}E\left(t\right)dB\left(t\right),\;t\geq 0$$

(10) $$dI\left(t\right)=\left(\alpha E\left(t\right)-\varepsilon_{2}I\left(t\right)-\delta I\left(t\right)-\mu I\left(t\right)\right)dt+\sigma_{3}I\left(t\right)dB\left(t\right),\;t\geq 0$$

(11) $$dR\left(t\right)=(\varepsilon_{1}E\left(t\right)+\varepsilon_{2}I\left(t\right)-\mu R\left(t\right))dt+\sigma_{4}R\left(t\right)dB\left(t\right),\;t\geq 0$$

where $\sigma_{i},\;i=1,2,3,4$ are the randomness of the model and B(t) is the Brownian motion.

### 3.3. Fundamental Properties

Consider $U\left(t\right)=\left(S\left(t\right),E\left(t\right),I\left(t\right),R\left(t\right)\right)$ and the norm:

(12) $$\left|U\left(t\right)\right|=\sqrt{S^{2}\left(t\right)+E^{2}\left(t\right)+I^{2}\left(t\right)+R^{2}\left(t\right)}$$

In addition, denote $C_{1}^{2,1}\left(R^{4}\times \left(0,\infty \right):R_{+}\right)$ as the families of all positive functions $V\left(U,t\right)$ defined on $R^{4}\times \left(0,\infty \right)$, respectively. Let the function be twice differentiable in U and once in t then,

(13) $$dU\left(t\right)=H_{1}\left(U,t\right)+K_{1}\left(U,t\right)dB\left(t\right)$$

In addition,$\;L=\frac{\partial }{\partial t}+\sum_{i=1}^{4}H_{1_{i}}\left(U,t\right)\frac{\partial }{\partial U_{i}}+\frac{1}{2}\sum_{i,j=1}^{4}(K_{1}^{T}\left(U,t\right)K_{1}{\left(U,t\right)}_{i,j}\times \frac{\partial^{2}}{\partial U_{i}\partial U_{j}})$.

If “L” acts on a function $U^{*}\epsilon C^{2,1}\left(R^{4}\times \left(0,\infty \right):R_{+}^{4}\right)$:

$$LU^{*}\left(U,t\right)\;=\;U_{t}^{*}\left(U,t\right)\;+\;U_{U}^{*}\left(U,t\right)H_{1}\left(U,t\right)\;+\;\frac{1}{2}Trace\left(K_{1}^{T}\left(U,t\right)U_{UU}^{*}\left(U,t\right)K_{1}\left(U,t\right)\right)$$

where T means Transportations.

**Definition** **3.** *Let* $B\left(t\right)$ *be a Brownian motion and* $I\left(t\right)$ *be an Ito drift-diffusion process that satisfies the stochastic differential equation:*

$$dI\left(t\right)=\mu \left(I\left(t\right),t\right)dt+\sigma \left(I\left(t\right),t\right)dB\left(t\right)\;$$

*If* $f\left(I,t\right)\in C^{4}\left(R^{4},\;R\right)$ *then* $f\left(I\left(t\right),t\right)$ *is also an Ito drift-diffusion process, which satisfies as follows:*

$$d\left(f\left(I\left(t\right),t\right)\right)=\frac{\partial f}{\partial t}\left(I\left(t\right),t\right)dt+f^{\prime }\left(\left(I\left(t\right),t\right)\right)dB\left(t\right)+\frac{1}{2}f^{\prime \prime }\left(\left(I\left(t\right),t\right)\right)dB{\left(t\right)}^{2}$$

**Theorem** **5.** *A unique solution (*$S\left(t\right),E\left(t\right),I\left(t\right),R\left(t\right)),\;t\geq 0$*of the system (8)–(11) lies in*$R_{+}^{4}$*with initial conditions:*$\left(S\left(0\right),E\left(0\right),I\left(0\right),R\left(0\right)\right)\epsilon \;R_{+}^{4}$.

**Proof.**  By Ito’s formula, (8)–(11) admit positive solution in the sense of unique local on $\left[0,\;\tau_{e}\right]$ while τe denotes the explosion time $\tau_{e}\;$ due to the local Lipschitz coefficients of the model.

Next, we shall prove that the system (8)–(11) model admits $\tau_{e}=\infty$.

Let $m_{0}=0$ be sufficiently large for S(0), E(0), I(0), and R(0) lying with the interval {$\frac{1}{m_{0}},m_{0}\}$.

A sequence at stopping times m≥0, defined as

(14) $$\tau_{m}=\inf\left\{\tau \epsilon \left[0,\tau_{e}\right]:S\left(t\right)\left(\frac{1}{m},m\right)or\;E\left(t\right)\left(\frac{1}{m},m\right)or\;I\left(t\right)\left(\frac{1}{m},m\right)or\;R\left(t\right)\left(\frac{1}{m},m\right)\right\}$$

where we set infφ=∞(φ is an empty set).

Since $\tau_{m}$ is increasing as m→∞

(15) $$\tau_{\infty }=\underset{m\to \infty }{\lim}\tau_{m}$$

Then, $\tau_{\infty }\leq \tau_{e}$. Now we wish to show that $\tau_{\infty }=\infty ,$ as desired.

(16) $$P\left(\tau_{\infty }\leq T\right)>a_{1},\;\forall m\geq m_{1},$$

(17) $$P\left(\tau_{m}\leq T\right)>a_{1},\;\forall m\geq m_{1}$$

Define a function $f:R_{+}^{4}\to R_{+}$ by:

(18) $$f\left(S,E,I,R\right)\;=\;\left(S-1-\ln S\right)+\left(E-1-\ln E\right)+\left(I-1-\ln I\right)+\left(R-1-\ln R\right)$$

Using Ito’s formula on (18), we have:

$$df\left(S,E,I,R\right)\;=\;\left(1-\frac{1}{S}\right)dS+\left(1-\frac{1}{E}\right)dE+\left(1-\frac{1}{I}\right)dI+\left(1-\frac{1}{R}\right)dR+\frac{\sigma_{1}^{2}+\sigma_{2}^{2}+\sigma_{3}^{2}+\sigma_{4}^{2}}{2}dt$$

$$\begin{array}{cc} df\left(S,E,I,R\right)\;=\; & \left(1-\frac{1}{S}\right)\left[A-\frac{\beta \left(1-n\lambda \gamma \right)\left(1-\gamma \lambda \alpha \right)}{N}IS-\mu S+\sigma_{1}SdB\left(t\right)\right]\\ & +\left(1-\frac{1}{E}\right)\left[\frac{\beta \left(1-n\lambda \gamma \right)\left(1-\gamma \lambda \alpha \right)}{N}IS-\alpha E-\varepsilon_{1}E-\mu E+\sigma_{2}EdB\left(t\right)\right]\\ & +\left(1-\frac{1}{I}\right)\left[\alpha E-\varepsilon_{2}I-\delta I-\mu I+\sigma_{3}IdB\left(t\right)\right]+\left(1-\frac{1}{R}\right)\left[\varepsilon_{1}E+\varepsilon_{2}I-\mu R+\sigma_{4}RdB\left(t\right)\right] \end{array}$$

(19) $$df\left(S,E,I,R\right)\;\leq \;\left[A+\frac{\sigma_{1}^{2}+\sigma_{2}^{2}+\sigma_{3}^{2}+\sigma_{4}^{2}}{2}\right]dt+\sigma_{1}SdB\left(t\right)+\sigma_{2}EdB\left(t\right)+\sigma_{3}IdB\left(t\right)+\sigma_{4}RdB\left(t\right)$$

For simplicity, we let $N_{1}=A+\frac{\sigma_{1}^{2}+\sigma_{2}^{2}+\sigma_{3}^{2}+\sigma_{4}^{2}}{2}$ and write Equation (19) as:

(20) $$df\left(S,E,I,R\right)\;\leq \;N_{1}dt+\left[\sigma_{1}S+\sigma_{2}E+\sigma_{3}I+\sigma_{4}R\right]dB\left(t\right)$$

The $N_{1}$ is a positive constant. By integrating Equation (20) from 0 to $\tau_{m}\Lambda \tau$,

(21) $$\overset{\tau_{m}\Lambda \tau }{\underset{0}{\int }}df\left(S,E,I,R\right)\;\leq \;\overset{\tau_{m}\Lambda \tau }{\underset{0}{\int }}N_{1}ds+\overset{\tau_{m}\Lambda \tau }{\underset{0}{\int }}\left[\sigma_{1}S+\sigma_{2}E+\sigma_{3}I+\sigma_{4}R\right]dB\left(s\right)$$

where $\tau_{m}\Lambda \tau$ = mini ($\tau_{m},T)$, then the expectation will be:

(22) $$EU^{*}(S\left(\tau_{m}\Lambda \tau \right),E\left(\tau_{m}\Lambda \tau \right),I\left(\tau_{m}\Lambda \tau \right),R\left(\tau_{m}\Lambda \tau \right)\;\leq \;U^{*}\left(S\left(0\right),E\left(0\right),I\left(0\right),R\left(0\right)\right)+N_{1}T$$

Set $\chi_{m}=\left\{\tau_{m}\leq T\right\}$ for $m>m_{1}$ and from Equation (15), we have $P(\chi_{m}\geq a_{1})$

For every $\chi_{1}\epsilon \chi_{m}$ there are some “I”s such that $U_{i}\left(\tau_{m},\chi_{1}\right)$ equals either m or $\frac{1}{m}$ for i = 1, 2, 3, 4. Hence,

$$U^{*}\left(S\left(\tau_{m},\chi_{1}\right),E\left(\tau_{m},\chi_{1}\right),I\left(\tau_{m},\chi_{1}\right),R\left(\tau_{m},\chi_{1}\right)\right)$$

For “I” less than min$\left(m-1-\ln m,\frac{1}{m}-1-\ln\frac{1}{m}\right\}$ then we obtain:

$$U^{*}\left(S\left(0\right),E\left(0\right),I\left(0\right),R\left(0\right)\right)+N_{1}T\geq E(I_{\chi }U^{*}\left(S\left(\tau_{m}\right),E\left(\tau_{m}\right),I\left(\tau_{m}\right),R\left(\tau_{m}\right)\right)\geq$$

(23) $$\left\{mini\{m-1-\ln m,\frac{1}{m}-1-\ln\frac{1}{m}\}\right\}$$

$I_{\chi }$ of $\chi_{m}$ represents the indicator functions. Letting m→∞ leads to the contradiction $\infty =U^{*}\left(S\left(0\right),E\left(0\right),I\left(0\right),R\left(0\right)\right)+N_{1}T<\infty$, as desired. □

## 4. Numerical Methods

This section deals with well-known methods like the stochastic Runge–Kutta, and the proposed stochastic NSFD method with the given non-negative initial conditions as follows:

### 4.1. Stochastic Runge–Kutta

The stochastic Runge–Kutta method could be developed on the system (12)–(15) as follows:

Stage 1

$$W_{1}=\Delta t_{n}\left(\Lambda -\frac{\beta \left(1-\eta \lambda \tau \right)\left(1-\gamma \lambda \sigma \right)I^{n}S^{n}}{N}-\mu S^{n}+\sigma_{1}S^{n}\Delta B_{n}\right)$$

$$X_{1}=\Delta t_{n}\left(\frac{\beta \left(1-\eta \lambda \tau \right)\left(1-\gamma \lambda \sigma \right)I^{n}S^{n}}{N}-\alpha E^{n}-\varepsilon_{1}E^{n}-\mu E^{n}+\sigma_{2}E^{n}\Delta B_{n}\right)$$

$$Y_{1}=\Delta t_{n}\left(\alpha E^{n}-\varepsilon_{2}I^{n}-\delta I^{n}-\mu I^{n}+\sigma_{3}I^{n}\Delta B_{n}\right)$$

$$Z_{1}=\Delta t_{n}\left(\varepsilon_{1}E^{n}+\varepsilon_{2}I^{n}-\mu R^{n}+\sigma_{4}R^{n}\Delta B_{n}\right)$$

Stage 2

$$W_{2}=\Delta t_{n}(\Lambda -\frac{\beta \left(1-\eta \lambda \tau \right)\left(1-\gamma \lambda \sigma \right)\left(I^{n}+\frac{Y_{1}}{2}\right)(S^{n}+\frac{W_{1}}{2})}{N}-\mu (S^{n}+\frac{W_{1}}{2})+\sigma_{1}(S^{n}+\frac{W_{1}}{2})\Delta B_{n})$$

$$X_{2}=\Delta t_{n}(\frac{\beta (1-\eta \lambda \tau )(1-\gamma \lambda \sigma )(I^{n}+\frac{Y_{1}}{2})(S^{n}+\frac{W_{1}}{2})}{N}-\alpha (E^{n}+\frac{X_{1}}{2})-\varepsilon_{1}(E^{n}+\frac{X_{1}}{2})-\mu (E^{n}+\frac{X_{1}}{2})+\sigma_{2}(E^{n}+\frac{X_{1}}{2})\Delta B_{n})$$

$$Y_{2}=\Delta t_{n}(\alpha (E^{n}+\frac{X_{1}}{2})-\varepsilon_{2}(I^{n}+\frac{Y_{1}}{2})-\delta (I^{n}+\frac{Y_{1}}{2})-\mu (I^{n}+\frac{Y_{1}}{2})+\sigma_{3}(I^{n}+\frac{Y_{1}}{2})\Delta B_{n})$$

$$Z_{2}=\Delta t_{n}(\varepsilon_{1}(E^{n}+\frac{X_{1}}{2})+\varepsilon_{2}(I^{n}+\frac{Y_{1}}{2})-\mu (R^{n}+\frac{Z_{1}}{2})+\sigma_{4}(R^{n}+\frac{Z_{1}}{2})\Delta B_{n})$$

Stage 3

$$W_{3}=\Delta t_{n}(\Lambda -\frac{\beta (1-\eta \lambda \tau )(1-\gamma \lambda \sigma )(I^{n}+\frac{Y_{2}}{2})(S^{n}+\frac{W_{2}}{2})}{N}-\mu (S^{n}+\frac{W_{2}}{2})+\sigma_{1}(S^{n}+\frac{W_{2}}{2})\Delta B_{n})$$

$$X_{3}=\Delta t_{n}(\frac{\beta (1-\eta \lambda \tau )(1-\gamma \lambda \sigma )(I^{n}+\frac{Y_{2}}{2})(S^{n}+\frac{W_{2}}{2})}{N}-\alpha (E^{n}+\frac{X_{2}}{2})-\varepsilon_{1}(E^{n}+\frac{X_{2}}{2})-\mu (E^{n}+\frac{X_{2}}{2})+\sigma_{2}(E^{n}+\frac{X_{2}}{2})\Delta B_{n})$$

$$Y_{3}=\Delta t_{n}(\alpha (E^{n}+\frac{X_{2}}{2})-\varepsilon_{2}(I^{n}+\frac{Y_{2}}{2})-\delta (I^{n}+\frac{Y_{2}}{2})-\mu (I^{n}+\frac{Y_{2}}{2})+\sigma_{3}(I^{n}+\frac{Y_{2}}{2})\Delta B_{n})$$

$$Z_{3}=\Delta t_{n}(\varepsilon_{1}(E^{n}+\frac{X_{2}}{2})+\varepsilon_{2}(I^{n}+\frac{Y_{2}}{2})-\mu (R^{n}+\frac{Z_{2}}{2})+\sigma_{4}(R^{n}+\frac{Z_{2}}{2})\Delta B_{n})$$

Stage 4

$$W_{4}=\Delta t_{n}(\Lambda -\frac{\beta (1-\eta \lambda \tau )(1-\gamma \lambda \sigma )(I^{n}+Y_{3})(S^{n}+W_{3})}{N}-\mu (S^{n}+W_{3})+\sigma_{1}(S^{n}+W_{3})\Delta B_{n})$$

$$X_{4}=\Delta t_{n}(\frac{\beta (1-\eta \lambda \tau )(1-\gamma \lambda \sigma )(I^{n}+Y_{3})(S^{n}+W_{3})}{N}-\alpha (E^{n}+X_{3})-\varepsilon_{1}(E^{n}+X_{3})-\mu (E^{n}+X_{3})+\sigma_{2}(E^{n}+X_{3})\Delta B_{n})$$

$$Y_{4}=\Delta t_{n}(\alpha (E^{n}+X_{3})-\varepsilon_{2}(I^{n}+Y_{3})-\delta (I^{n}+Y_{3})-\mu (I^{n}+Y_{3})+\sigma_{3}(I^{n}+Y_{3})\Delta B_{n})$$

$$Z_{4}=\Delta t_{n}(\varepsilon_{1}(E^{n}+X_{3})+\varepsilon_{2}(I^{n}+Y_{3})-\mu (R^{n}+Z_{3})+\sigma_{4}(R^{n}+Z_{3})\Delta B_{n})$$

**Figure 4:**
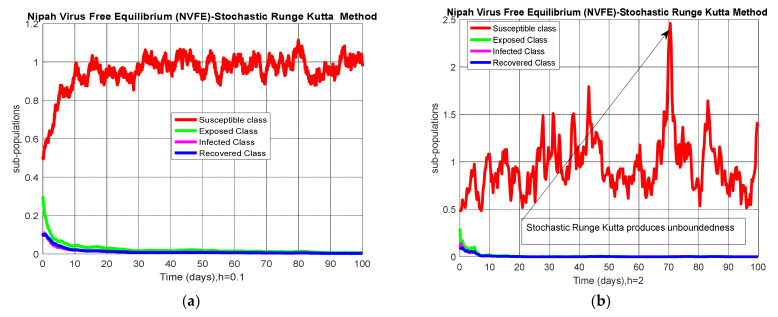

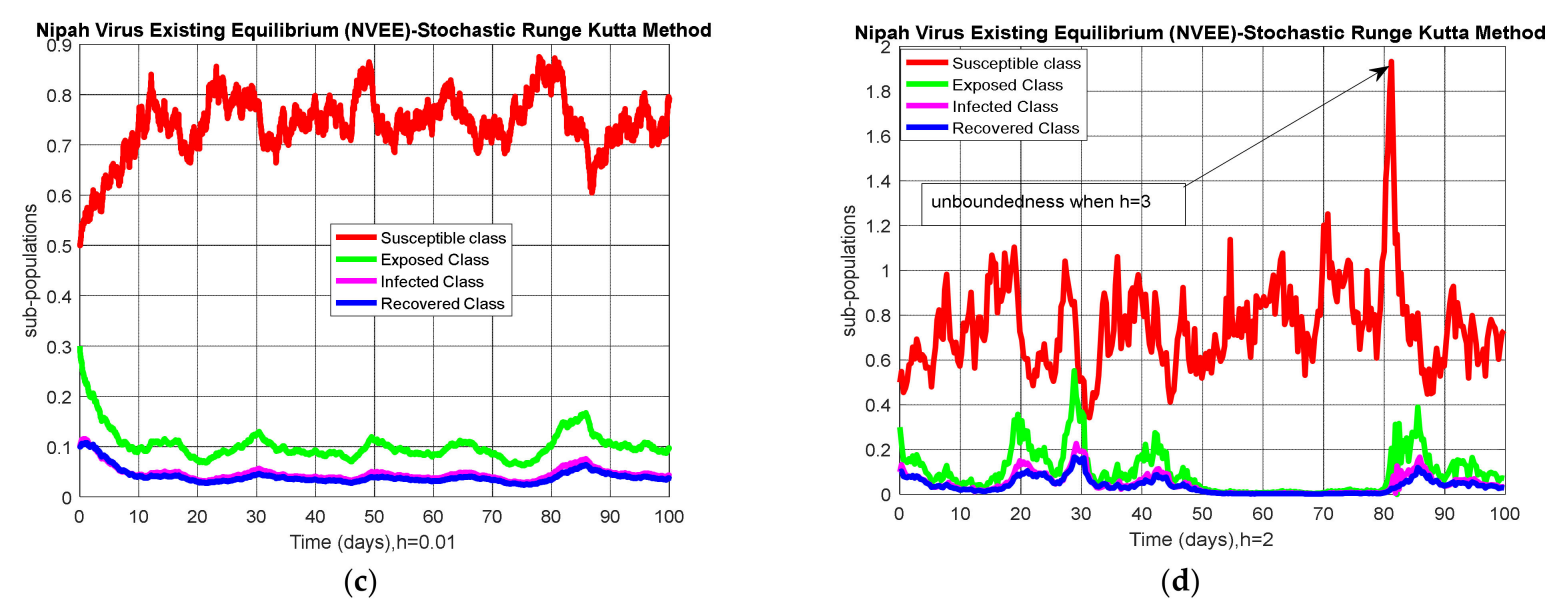
(**a**) Sub-population for NVFE at h=0.01; (**b**) sub-population for NVFE at h=2; (**c**) sub-population for NVEE at h=0.01; (**d**) sub-population for NVEE at h=2.

Final stage:

(24) $$S^{n+1}=S^{n}+\frac{1}{6}(W_{1}+2W_{2}+2W_{3}+W_{4})$$

(25) $$E^{n+1}=E^{n}+\frac{1}{6}(X_{1}+2X_{2}+2X_{3}+X_{4})$$

(26) $$I^{n+1}=I^{n}+\frac{1}{6}(Y_{1}+2Y_{2}+2Y_{3}+Y_{4})$$

(27) $$R^{n+1}=R^{n}+\frac{1}{6}(Z_{1}+2Z_{2}+2Z_{3}+Z_{4})$$

where $“\Delta t_{n}”$ is any time step size. The simulations of the stochastic Runge–Kutta method for Nipah virus-free equilibrium (NVFE) and Nipah virus-existing equilibrium (NVEE) by using the data presented in Table 2 are shown in Figure 4.

### 4.2. Stochastic NSFD

The stochastic NSFD could be developed for the system (8)–(11) as follows:

(28) $$S^{n+1}=\frac{S^{n}+\Delta t_{n}\Lambda +h\sigma_{1}S^{n}\Delta B_{n}}{\left(1+\frac{\Delta t_{n}\beta (1-\eta \lambda \tau )(1-\gamma \lambda \sigma )I^{n}}{N}+\Delta t_{n}\mu \right)}$$

(29) $$E^{n+1}=\frac{E^{n}+\frac{\Delta t_{n}\beta (1-\eta \lambda \tau )(1-\gamma \lambda \sigma )I^{n}S^{n}}{N}+\Delta t_{n}\sigma_{1}E^{n}\Delta B_{n}}{\left(1+\Delta t_{n}(\alpha +\varepsilon_{1}+\mu )\right)}$$

(30) $$I^{n+1}=\frac{I^{n}+\Delta t_{n}\alpha E^{n}+\Delta t_{n}\sigma_{2}I^{n}\Delta B_{n}}{\left(1+\Delta t_{n}(\varepsilon_{2}+\delta +\mu )\right)}$$

(31) $$R^{n+1}=\frac{R^{n}+\Delta t_{n}E^{n}+\Delta t_{n}\varepsilon_{2}I^{n}+\Delta t_{n}\sigma_{2}R^{n}\Delta B_{n}}{\left(1+\Delta t_{n}\mu \right)}$$

where $“\Delta t_{n}”$ is anytime step size.

### 4.3. Stability Analysis

**Theorem** **6.** *The stochastic NSFD method is stable if the eigenvalues of Equations (28)–(31) lie in the same unit circle for any*n≥0.

**Proof.**  Considering the functions from the system (28)–(31), we have

$$A=\frac{S+\Delta t_{n}\Lambda }{\left(1+\frac{\Delta t_{n}\beta (1-\eta \lambda \tau )(1-\gamma \lambda \sigma )I}{N}+\Delta t_{n}\mu \right)},\;B=\frac{E+\frac{\Delta t_{n}\beta (1-\eta \lambda \tau )(1-\gamma \lambda \sigma )IS}{N}}{\left(1+\Delta t_{n}(\alpha +\varepsilon_{1}+\mu \right)},\;C=\frac{I+\Delta t_{n}\alpha E}{\left(1+\Delta t_{n}(\varepsilon_{2}+\delta +\mu )\right)},\;D=\frac{R+\Delta t_{n}\varepsilon_{1}E+\Delta t_{n}\varepsilon_{2}I}{\left(1+\Delta t_{n}\mu \right)}.$$

The element of Jacobian matrix is as follows:

$$\frac{\partial A}{\partial S}=\frac{1}{\left(1+\frac{\Delta t_{n}\beta (1-\eta \lambda \tau )(1-\gamma \lambda \sigma )I}{N}+\Delta t_{n}\mu \right)},\;\frac{\partial A}{\partial E}=0,\;\frac{\partial A}{\partial I}=\frac{N\Delta t_{n}\beta (S+\Delta t_{n}\Lambda )(1-\eta \lambda \tau )(1-\gamma \lambda \sigma )}{{\left\{N+\Delta t_{n}\beta (1-\eta \lambda \tau )(1-\gamma \lambda \sigma )I+\Delta t_{n}\mu N\right\}}^{2}},\;\frac{\partial A}{\partial R}=0$$

$$\frac{\partial B}{\partial S}=\frac{\Delta t_{n}\beta (1-\eta \lambda \tau )(1-\gamma \lambda \sigma )I}{N(1+\Delta t_{n}(\alpha +\varepsilon_{1}+\mu ))},\;\frac{\partial B}{\partial E}=\frac{I}{N(1+\Delta t_{n}(\alpha +\varepsilon_{1}+\mu ))},\;\frac{\partial B}{\partial I}=\frac{\Delta t_{n}\beta (1-\eta \lambda \tau )(1-\gamma \lambda \sigma )S}{N(1+\Delta t_{n}(\alpha +\varepsilon_{1}+\mu )},\;\frac{\partial B}{\partial R}=0,$$

$$\frac{\partial C}{\partial S}=0,\;\frac{\partial C}{\partial E}=\frac{\Delta t_{n}\alpha }{\left(1+\Delta t_{n}(\varepsilon_{2}+\delta +\mu )\right)},\;\frac{\partial C}{\partial I}=\frac{1}{\left(1+\Delta t_{n}(\varepsilon_{2}+\delta +\mu )\right)},\;\frac{\partial C}{\partial R}=0$$

$$\frac{\partial D}{\partial S}=0,\;\frac{\partial D}{\partial E}=\frac{\Delta t_{n}\varepsilon_{1}}{\left(1+\Delta t_{n}\mu \right)},\;\frac{\partial D}{\partial I}=\frac{\Delta t_{n}\varepsilon_{2}}{\left(1+\Delta t_{n}\mu \right)},\;\frac{\partial D}{\partial R}=\frac{1}{\left(1+\Delta t_{n}\mu \right)}$$

$$J=\left[\begin{array}{cccc} \frac{1}{\left(1+\frac{\Delta t_{n}\beta \left(1-\eta \lambda \tau \right)\left(1-\gamma \lambda \sigma \right)I}{N}+\Delta t_{n}\mu \right)} & 0 & \frac{-N\Delta t_{n}\beta \left(S+\Delta t_{n}\Lambda \right)\left(1-\eta \lambda \tau \right)\left(1-\gamma \lambda \sigma \right)}{{\left\{N+\Delta t_{n}\beta \left(1-\eta \lambda \tau \right)\left(1-\gamma \lambda \sigma \right)I+\Delta t_{n}\mu N\right\}}^{2}} & 0\\ \frac{\Delta t_{n}\beta \left(1-\eta \lambda \tau \right)\left(1-\gamma \lambda \sigma \right)I}{N\left(1+\Delta t_{n}(\alpha +\varepsilon_{1}+\mu \right))} & \frac{1}{\left(1+\Delta t_{n}\left(\alpha +\varepsilon_{1}+\mu \right)\right)} & \frac{\Delta t_{n}\beta \left(1-\eta \lambda \tau \right)\left(1-\gamma \lambda \sigma \right)S}{N\left(1+\Delta t_{n}\left(\alpha +\varepsilon_{1}+\mu \right)\right)} & 0\\ 0 & \frac{\Delta t_{n}\alpha }{\left(1+\Delta t_{n}(\varepsilon_{2}+\delta +\mu \right))} & \frac{1}{\left(1+\Delta t_{n}(\varepsilon_{2}+\delta +\mu \right))} & 0\\ 0 & \frac{\Delta t_{n}\varepsilon_{1}}{\left(1+\Delta t_{n}\mu \right)} & \frac{\Delta t_{n}\varepsilon_{2}}{\left(1+\Delta t_{n}\mu \right)} & \frac{1}{\left(1+\Delta t_{n}\mu \right)} \end{array}\right]$$

$$\left|\begin{array}{c} J\left(\frac{\Lambda }{\mu },0,0,0\right) \end{array}\right|=\left|\begin{array}{cccc} \frac{N}{N+\Delta t_{n}\beta \left(1-\eta \lambda \tau \right)\left(1-\gamma \lambda \sigma \right)\left(0\right)+\Delta t_{n}\mu N} & 0 & \frac{-N\Delta t_{n}\beta \left(\frac{\Lambda }{\mu }+\Delta t_{n}\Lambda \right)\left(1-\eta \lambda \tau \right)\left(1-\gamma \lambda \sigma \right)}{{\left\{N+\Delta t_{n}\mu N\right\}}^{2}} & 0\\ 0 & \frac{1}{\left(1+\Delta t_{n}\left(\alpha +\varepsilon_{1}+\mu \right)\right)} & \frac{h\beta \left(1-\eta \lambda \tau \right)\left(1-\gamma \lambda \sigma \right)\frac{\Lambda }{\mu }}{N\left(1+\Delta t_{n}\left(\alpha +\varepsilon_{1}+\mu \right)\right)} & 0\\ 0 & \frac{\Delta t_{n}\alpha }{\left(1+\Delta t_{n}(\varepsilon_{2}+\delta +\mu \right))} & \frac{1}{\left(1+\Delta t_{n}(\varepsilon_{2}+\delta +\mu \right))} & 0\\ 0 & \frac{\Delta t_{n}\varepsilon_{1}}{\left(1+\Delta t_{n}\mu \right)} & \frac{\Delta t_{n}\varepsilon_{2}}{\left(1+\Delta t_{n}\mu \right)} & \frac{1}{\left(1+\Delta t_{n}\mu \right)} \end{array}\right|=0$$

$$\left|\begin{array}{c} J\left(\frac{\Lambda }{\mu },0,0,0\right) \end{array}\right|=\left|\begin{array}{cccc} \frac{N}{N+\Delta t_{n}\beta \left(1-\eta \lambda \tau \right)\left(1-\gamma \lambda \sigma \right)\left(0\right)+\Delta t_{n}\mu N} & 0 & \frac{-N\Delta t_{n}\beta \left(\frac{\Lambda }{\mu }+\Delta t_{n}\Lambda \right)\left(1-\eta \lambda \tau \right)\left(1-\gamma \lambda \sigma \right)}{{\left\{N+\Delta t_{n}\mu N\right\}}^{2}} & 0\\ 0 & \frac{1}{\left(1+\Delta t_{n}\left(\alpha +\varepsilon_{1}+\mu \right)\right)} & \frac{h\beta \left(1-\eta \lambda \tau \right)\left(1-\gamma \lambda \sigma \right)\frac{\Lambda }{\mu }}{N\left(1+\Delta t_{n}\left(\alpha +\varepsilon_{1}+\mu \right)\right)} & 0\\ 0 & \frac{\Delta t_{n}\alpha }{\left(1+\Delta t_{n}(\varepsilon_{2}+\delta +\mu \right))} & \frac{1}{\left(1+\Delta t_{n}(\varepsilon_{2}+\delta +\mu \right))} & 0\\ 0 & \frac{\Delta t_{n}\varepsilon_{1}}{\left(1+\Delta t_{n}\mu \right)} & \frac{\Delta t_{n}\varepsilon_{2}}{\left(1+\Delta t_{n}\mu \right)} & \frac{1}{\left(1+\Delta t_{n}\mu \right)} \end{array}\right|=0$$

$$\left|\begin{array}{c} J\left(\frac{\Lambda }{\mu },0,0,0\right)-\lambda^{*} \end{array}\right|=\left|\begin{array}{cccc} \frac{1}{\left(1+\Delta t_{n}\mu \right)}-\lambda^{*} & 0 & \frac{-N\Delta t_{n}\beta \left(\frac{\Lambda }{\mu }+\Delta t_{n}\Lambda \right)\left(1-\eta \lambda \tau \right)\left(1-\gamma \lambda \sigma \right)}{N^{2}{\left\{1+\Delta t_{n}\mu \right\}}^{2}} & 0\\ 0 & \frac{1}{\left(1+\Delta t_{n}(\varepsilon_{2}+\delta +\mu \right))}-\lambda^{*} & \frac{\Delta t_{n}\beta \Lambda \left(1-\eta \lambda \tau \right)\left(1-\gamma \lambda \sigma \right)}{\mu N\left(1+\Delta t_{n}\left(\alpha +\varepsilon_{1}+\mu \right)\right)} & 0\\ 0 & \frac{\Delta t_{n}\alpha }{\left(1+\Delta t_{n}(\varepsilon_{2}+\delta +\mu \right))} & \frac{1}{\left(1+\Delta t_{n}(\varepsilon_{2}+\delta +\mu \right))}-\lambda^{*} & 0\\ 0 & \frac{\Delta t_{n}\varepsilon_{1}}{\left(1+\Delta t_{n}\mu \right)} & \frac{\Delta t_{n}\varepsilon_{2}}{\left(1+\Delta t_{n}\mu \right)} & \frac{1}{\left(1+\Delta t_{n}\mu \right)}-\lambda^{*} \end{array}\right|=0$$

$$\left|\begin{array}{c} J\left(\frac{\Lambda }{\mu },0,0,0\right)-\lambda^{*} \end{array}\right|=\left|\begin{array}{ccc} \frac{1}{\left(1+\Delta t_{n}\mu \right)}-\lambda^{*} & 0 & \frac{-N\Delta t_{n}\beta \left(\frac{\Lambda }{\mu }+\Delta t_{n}\Lambda \right)\left(1-\eta \lambda \tau \right)\left(1-\gamma \lambda \sigma \right)}{N^{2}{\left\{1+\Delta t_{n}\mu \right\}}^{2}}\\ 0 & \frac{1}{\left(1+\Delta t_{n}(\varepsilon_{2}+\delta +\mu \right))}-\lambda^{*} & \frac{\Delta t_{n}\beta \Lambda \left(1-\eta \lambda \tau \right)\left(1-\gamma \lambda \sigma \right)}{\mu N\left(1+\Delta t_{n}\left(\alpha +\varepsilon_{1}+\mu \right)\right)}\\ 0 & \frac{\Delta t_{n}\alpha }{\left(1+\Delta t_{n}(\varepsilon_{2}+\delta +\mu \right))} & \frac{1}{\left(1+\Delta t_{n}(\varepsilon_{2}+\delta +\mu \right))}-\lambda^{*} \end{array}\right|=0$$

$$\frac{1}{\left(1+\Delta t_{n}\mu \right)}-\lambda^{*}=0$$

$$\lambda^{*}=\frac{1}{\left(1+\Delta t_{n}\mu \right)}<1$$

$$\left|\begin{array}{c} J\left(\frac{\Lambda }{\mu },0,0,0\right)-\lambda^{*} \end{array}\right|=\left|\begin{array}{ccc} \omega -\lambda^{*} & 0 & \frac{-\Psi \Lambda \left(\frac{1}{\mu }+\Delta t_{n}\right)}{N\omega^{2}}\\ 0 & \theta -\lambda^{*} & \Psi \Lambda \theta \Omega \\ 0 & \Delta t_{n}\alpha \phi & \phi -\lambda^{*} \end{array}\right|=0$$

where $\frac{1}{\left(1+\Delta t_{n}\left(\alpha +\varepsilon_{1}+\mu \right)\right)}=\theta$, $\frac{1}{\left(1+\Delta t_{n}(\varepsilon_{2}+\delta +\mu \right))}=\phi$, $\Delta t_{n}\beta \left(1-\eta \lambda \tau \right)\left(1-\gamma \lambda \sigma \right)=\Psi$, $\frac{1}{1+\Delta t_{n}\mu }=\omega$, $\frac{1}{\mu N}=\Omega$

$$\lambda^{*}{}^{2}-\theta \lambda^{*}-\phi \lambda^{*}+\theta \phi -\Delta t_{n}\alpha \phi \Psi \Lambda \theta \Omega \;=\;0$$

$$\lambda^{*}{}^{2}-(\theta +\phi )\lambda^{*}+\theta \phi \left(1-\Delta t_{n}\alpha \Psi \Lambda \Omega \right)\;=\;0$$

□

**Lemma** **1.** *For the quadratic equation:* $\lambda^{2}\;-\;P_{1}\lambda +P_{2}=0\;$ *,* $\left|\lambda_{i}\right|<1,\;i=1,\;2$ *, 3, if and only if the following conditions are satisfied:*

*(i)*  $1+P_{1}+P_{2}>0$.
*(ii)*  $1-P_{1}+P_{2}>0$.
*(iii)*  $P_{2}<1$.

**Proof.**  The proof is straightforward. □

### 4.4. Comparison Section

**Figure 5:**
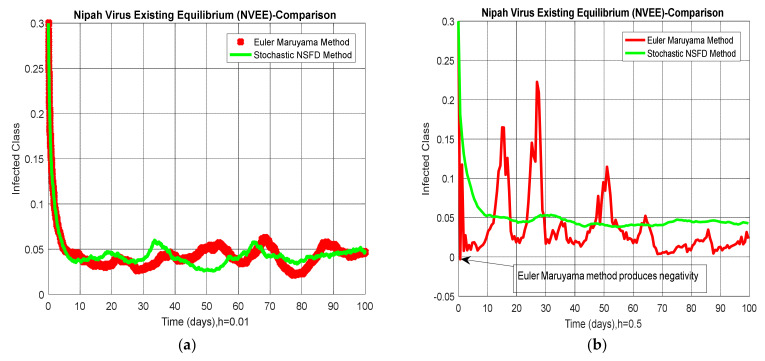

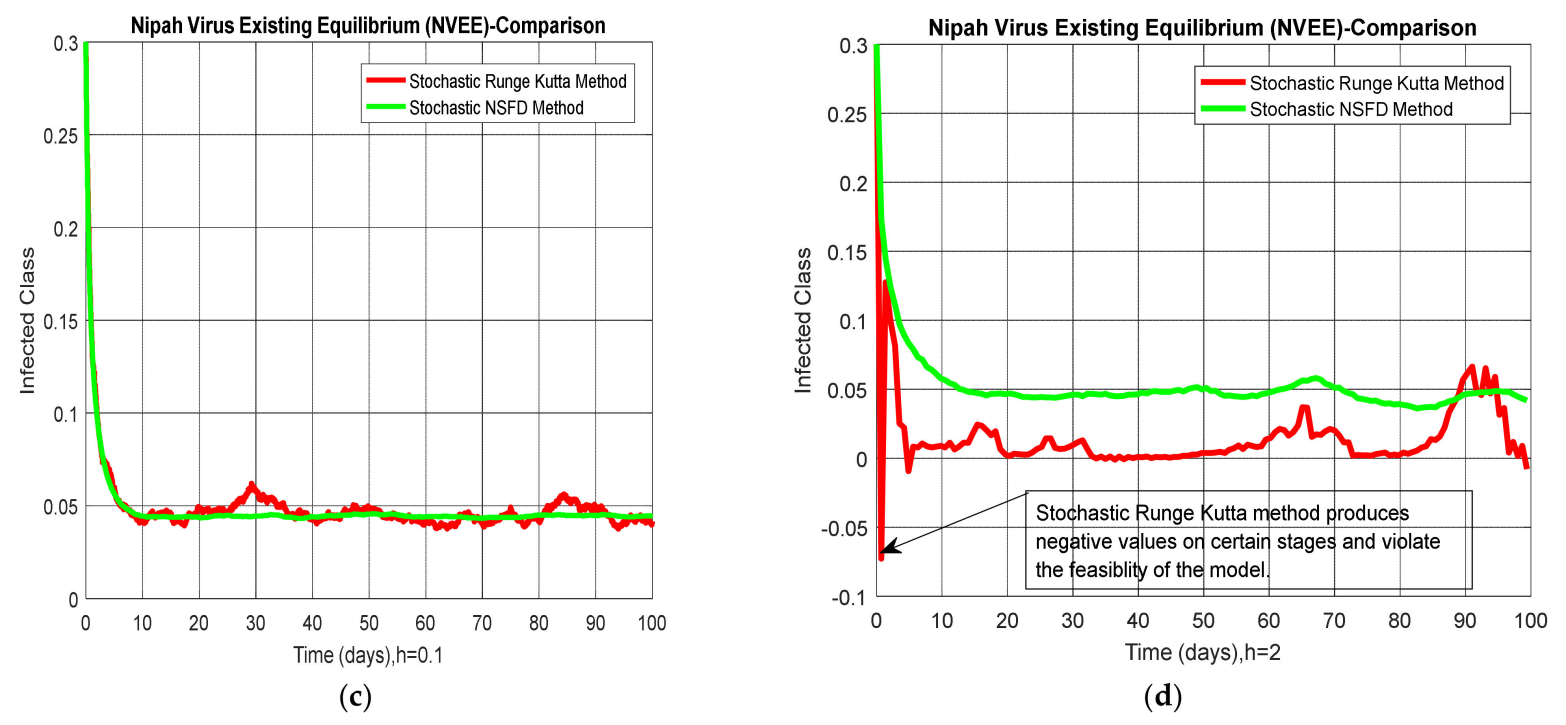
(**a**) Convergent behavior of Euler–Maruyama with NSFD at h=0.01; (**b**) divergent behavior of Euler–Maruyama with NSFD at h=0.5; (**c**) convergent behavior of stochastic Runge–Kutta with NSFD at h=0.1; (**d**) divergent behavior of stochastic Runge–Kutta with NSFD at h=2.

A comparison of the stochastic NSFD method with other stochastic numerical methods is presented. It is easy to see that other stochastic numerical methods conditionally converge or diverge with larger time step values by looking at the numerical solutions, as shown in Figure 5.

## 5. Results and Discussion

Through this study, we investigated the transmission dynamics of the Nipah virus in humans. The whole manuscript comprises three Sections. Modeling, terminology related to epidemiology, and Nipah virus are the critical points of Section 1. Analysis of the model is investigated in Section 2. Computational analysis, including well-known methods, is presented in Section 3. Mostly, methods are valid for only tiny time step sizes but inappropriately flop for huge time step sizes like Euler–Maruyama and stochastic Runge–Kutta methods. Our proposed scheme (SNSFD) remains convergent for anytime step sizes like *h* = 100. Furthermore, Table 3 shows the comparison for convergence behavior of different numerical schemes. The standard finite difference schemes like Euler–Maruyama and Stochastic RK4 are highly dependent on step size h and show divergence when h increases from a specific value. The proposed stochastic NSFD method is independent of discretization parameter h and exhibits the convergence for even enormous values of h like h = 100. This feature of the proposed scheme shows a significant advantage over the other methods in terms of computational efficiency and unconditional convergence.

## 6. Conclusions

The stochastic non-standard finite difference scheme is designed for the communication dynamics of the Nipah virus. Unfortunately, the methods mentioned earlier, like Euler–Maruyama and stochastic Runge–Kutta of order 4th, are unsuitable because they depend on time step size. Thus, Euler–Maruyama and stochastic Runge–Kutta are tentatively convergent. When we increase the time step size, the graph of Euler–Maruyama and stochastic Runge–Kutta gives variation in results from time to time that they display as divergent. Furthermore, the existing numerical methods did not preserve the structure of the continuous model. Thus, these are the gaps in the literature that need to be filled. For this reason, we have introduced the non-standard finite difference method, which preserves the actual structure of the continuous model, such as positivity, boundedness, and dynamical consistency. The new well-known numerical scheme—such as the stochastic non-standard finite difference scheme—is independent of time step size. The SNSFD scheme is a comfortable tool on behalf of dynamical properties like stability, positivity, and boundedness and shows the exact behavior of the continuous model. In the future, we will extend the idea used in this work to different types of modelling, including spatiotemporal, fractional, fractal fractional, and delay problems of dynamical systems.

## Figures and Tables

**Table 1:** Transition probabilities of Nipah virus epidemic model.

Transition	Probabilities
${\left(\Delta C\right)}_{1}\;=\;{\left[1\;0\;0\;0\right]}^{T}$	$P_{1}\;=\;\left(\Lambda \right)\Delta t$
${\left(\Delta C\right)}_{2}\;=\;{\left[-1\;1\;0\;0\right]}^{T}$	$P_{2}\;=\;\left(\frac{\beta \left(1-\eta \lambda \tau \right)\left(1-\gamma \lambda \sigma \right)IS}{N}\right)\Delta t$
${\left(\Delta C\right)}_{3}\;=\;{\left[-1\;0\;0\;0\right]}^{T}$	$P_{3}\;=\;\left(\Lambda S\right)\Delta t$
${\left(\Delta C\right)}_{4}\;=\;{\left[0\;-1\;1\;0\right]}^{T}$	$P_{4}\;=\;\left(\alpha E\right)\Delta t$
${\left(\Delta C\right)}_{5}\;=\;{\left[0\;-1\;0\;1\right]}^{T}$	$P_{5}\;=\;\left(\varepsilon_{1}E\right)\Delta t$
${\left(\Delta C\right)}_{6}\;=\;{\left[0\;-1\;0\;0\right]}^{T}$	$P_{6}\;=\;\left(\mu E\right)\Delta t$
${\left(\Delta C\right)}_{7}\;=\;{\left[0\;0\;-1\;1\right]}^{T}$	$P_{7}\;=\;\left(\varepsilon_{2}I\right)\Delta t$
${\left(\Delta C\right)}_{8}\;=\;{\left[0\;0\;-1\;0\right]}^{T}$	$P_{8}\;=\;\left(\delta I\right)\Delta t$
${\left(\Delta C\right)}_{9}\;=\;{\left[0\;0\;-1\;0\right]}^{T}$	$P_{9}\;=\;\left(\mu I\right)\Delta t$
${\left(\Delta C\right)}_{10}\;=\;{\left[0\;0\;0\;-1\right]}^{T}$	$P_{10}\;=\;\left(\mu R\right)\Delta t$

**Table 2:** Values of Parameter (Fitted data).

Parameters	Values
Λ	0.5
δ	0.76
α	0.60
$\varepsilon_{1}$	0.15
$\varepsilon_{2}$	0.09
β	≥2.75
γ	≥0
λ	0.85
k	0.1
η	≥0
μ	0.5
σ	0.90

**Table 3:** Comparison analysis of methods at different values of h.

h	Euler–Maruyama	Stochastic Runge–Kutta	Stochastic NSFD
0.01	EE = Convergence DFE = Convergence	EE = Convergence DFE = Convergence	Convergence
0.1	EE = Convergence DFE = Convergence	EE = Convergence DFE = Convergence	Convergence
1	EE = Divergence DFE = Divergence	EE = Divergence DFE = Divergence	Convergence
10	Divergence (method failed)	Divergence	Convergence
100	Divergence (method failed)	Divergence	Convergence
1000	Divergence (method failed)	Divergence	Convergence

## Data Availability

All of the necessary data and the implementation details have been included in the manuscript.
